# Does COVID-19 Impact Less on Post-stroke Aphasia? This Is Not the Case

**DOI:** 10.3389/fpsyg.2020.564717

**Published:** 2020-11-30

**Authors:** Francesca Pisano, Alberto Giachero, Cristian Rugiero, Melanie Calati, Paola Marangolo

**Affiliations:** ^1^Dipartimento di Studi Umanistici, University of Naples Federico II, Naples, Italy; ^2^Aphasia Experimental Laboratory – Fondazione Carlo Molo Onlus, Turin, Italy; ^3^Dipartimento di Psicologia, University of Turin, Turin, Italy; ^4^IRCCS Fondazione Santa Lucia, Rome, Italy

**Keywords:** COVID-19, aphasia (language), stroke, depression, anxiety, psychosocial well being

## Abstract

**Background:**

The coronavirus disease 2019 (COVID-19) pandemic has greatly affected people’s mental health resulting in severe psychological consequences. One of the leading causes of long-term disability worldwide is aphasia. The language changes experienced by a person with aphasia (PWA) often have a sudden and long-lasting negative impact on social interaction, quality of life, and emotional wellbeing. The main aim of this study was to investigate the impact of COVID-19 on the different psychosocial dimensions which affect PWA.

**Methods:**

This retrospective study included 73 PWA and 81 elderly matched controls. All patients were in the chronic phase. They were all discharged from rehabilitation services, which left them with different degrees of language deficits (i.e., severe vs. mild vs. moderate). All participants were administered the hospital anxiety and depression scale (HADS) through an online survey. PWA also took part in the stroke and aphasia quality of life scale questionnaire (SAQOL-39).

**Results:**

Although the comparison between two different time points [one month before (T0) and one month after the lockdown (T1)] led to a significant increase in depression and anxiety symptoms in both groups (PWA vs. control), lower rates of depression and anxiety were found in PWA compared to the healthy group. Significant deterioration was also present in PWA in the communication and psychosocial scales of the SAQOL-39 test, which correlated with the observed changes in the psychological domains. Interestingly, the results were not significantly influenced by the degree of aphasia severity. Similarly, in both groups, none of the demographic variables (gender, age, and educational level) significantly affected the scores in the different subscales.

**Conclusions:**

This evidence which, at first glance, seems to suggest that PWA have been partially spared from the impact of COVID-19, actually masks a dramatic situation that has always characterized this population. Indeed, given that PWA already live in a state of social isolation and emotional instability, these conditions might have, paradoxically, limited the effects of the coronavirus. However, as our results showed a deterioration in the emotional state and communication skills of our patients, possible solutions are discussed in order to prevent further decline of their cognitive abilities.

## Introduction

First identified in China in late 2019, the coronavirus disease 2019 (COVID-19) emergency has become a global pandemic, spreading all over the world within a short period of time, including Italy (Şencan and Kuzi, 2020; World Health Organization [WHO], 2020). On March 8, 2020, the Italian Government implemented extraordinary measures to limit viral transmission, restricting movement in all regions of Italy using stay-at-home orders and lockdowns, limiting physical contact between people with the months-long suspension of commercial, educational, and social activities (Soraci et al., 2020). Given the unknown causes of the infection, the poor knowledge of its transmission, the unpredictability of the duration, and the high risk of mortality, the outbreak of COVID-19 led to severe physical and psychological consequences for people’s health (Brooks et al., 2020; Kang et al., 2020; Li et al., 2020; Moccia et al., 2020). Indeed, all over the world people are still mostly affected by negative emotions (i.e., depressive symptoms, anxiety, high stress levels, confusion, post-traumatic stress disorders, and insomnia) and negative cognitive assessment for self-protection (i.e., feelings of helplessness and fear of falling sick or dying), breaking the balance of daily activities, and decreasing the perception of wellbeing (Brooks et al., 2020; Kang et al., 2020; Li et al., 2020; Moccia et al., 2020). The work of Santini and collaborators (Santini et al., 2020) also reported a strong correlation between social isolation and high rates of anxiety and depression in both healthy young and older adults.

Since COVID-19 has resulted in serious negative psychological consequences in different populations, we wonder whether coronavirus has also dramatically affected people with brain damage, and, in particular, persons with post-stroke aphasia (PWA).

As is well known, stroke is one of the leading causes of long-term disability worldwide (Feigin et al., 2015). After a stroke, survivors can experience a wide range of difficulties, among them aphasia, which is one of the most serious socially disabling consequences. Aphasia, the loss of ability to produce and/or to understand language, manifests itself in about one-third of left brain-damaged people (30% of acute vs. 10–20% of chronic stroke patients, Ali et al., 2015). The aphasic symptoms are heterogeneous, varying in terms of severity and degree of involvement across the modalities of language, including the expression and comprehension of speech, reading, and writing. Variation in the severity of expressive impairments, for example, may range from the patient’s occasional inability to find the correct word to telegraphic and very reduced speech output. Thus, PWA experience frustration and depression since their exclusion from language-dependent activities has strong implications for many aspects of their emotional condition and social status. Indeed, the language changes experienced by PWA often have sudden and long-lasting negative impacts on friendships, social engagements, quality of life, and psychological wellbeing (Engelter et al., 2006; Spaccavento et al., 2014; Mattioli, 2019). Less curiosity and less emotional stability with anxiety and depression, distress, social exclusion, and loss of autonomy are frequently reported symptoms in aphasic people (Kouwenhoven et al., 2011; Ayerbe et al., 2014; Hackett and Pickles, 2014; Feigin et al., 2015; Robinson and Jorge, 2016; Mitchell et al., 2017; Kirkevold et al., 2018; Torres-Prioris et al., 2019). In particular, regardless of the degree of language impairment, the patient tends to isolate him/herself and to have less intrinsic motivation in taking part in language rehabilitation programs since he/she feels oppressed by negative feelings (Cott et al., 2007; Woodman et al., 2014; Foley et al., 2019; Hjelle et al., 2019). Accordingly, several studies have already pointed out that PWA have a quality of life worse than non-aphasic patients (Spaccavento et al., 2014; Wray and Clarke, 2017; Kirkevold et al., 2018; Hjelle et al., 2019).

Considering that COVID-19 has dramatically maximized the risk of social isolation and associated depression, in the present study, we wanted to investigate if the psychosocial difficulties experienced by PWA would have worsened due to coronavirus. Indeed, language problems limit the use of digital media (i.e., cellular and/or social networks) in order to maintain social contact. Thus, aphasic patients could not even rely on these social means, as other stroke or healthy people do, to compensate for the lack of social interaction imposed by the coronavirus.

As far as we know, to date, no study has explored the effects of COVID-19 on patients with chronic post-stroke aphasia.

In this exploratory retrospective study, we investigated the psychological impact of the COVID-19 outbreak on a group of chronic PWA matched with a control group of elderly participants by comparing their psychological state one month before (T0) to one month after lockdown.

## Design and Participants

The study design was an interview-based psychometric study. Seventy-three patients with post-stroke aphasia were recruited from two different speech and language therapy (SLT) service providers in Italy, one in Rome and the other in Turin.

All patients were already discharged from rehabilitation services before the COVID-19 emergency. They were all native Italian speakers with right premorbid manual dominance, affected by a single left hemispheric stroke occurring at least one year prior to experimentation. They were all cared for by a caregiver and lived at home before the stroke. No patients presented a history of severe cognitive decline, mental health problems, alcohol or drug abuse, head injury, or tumoral lesions. None of the participants had received structured language therapy for at least 6 months before the time of inclusion in the study in order to prevent confounding therapy effects. A group of 81 age- and education-matched elderly individuals were also recruited to serve as normal controls. They were all retired from work and they mostly stayed at home.

Persons with post-stroke aphasia had a mean age of 64.52 years (SD ± 9.85), 41 (56%) were male and 32 (44%) were female. The mean educational level was 11.88 (SD ± 3.85). For the control group, the mean age was 68.11 years (SD ± 10.08), 38 (47%) were male and 43 (53%) were female. The mean educational level was 11.27 (SD ± 4.07). The two groups did not significantly differ in age, educational level, and gender from each other. According to their age, all participants were subdivided into three groups: (1) 40–55 years (12 PWA, 11 controls), (2) 55–70 years (36 PWA, 34 controls), and (3) 70–85 years (25 PWA, 36 controls). According to the educational level, three different groups were identified: (1) 5–8 years (26 PWA, 37 controls), (2) 8–13 years (30 PWA, 26 controls), and (3) 13–17 years (17 PWA, 18 controls).

### Ethical Approval

An ethical review and approval were not required for the study on human participants in accordance with the local legislation, institutional requirements, and the legislation governing the psychologist profession (L.56/89). Written informed consent to participate in the study was provided by the patients.

### Procedure and Outcome Measurements

We used the Esame del Linguaggio II (Ciurli et al., 1996) and the Aachen Aphasia Test (AAT, Luzzatti et al., 1996) to screen for aphasia. According to their AAT score (Luzzatti et al., 1996), all patients were divided into three groups: 30 with severe/global aphasia (13 males and 17 females; age 65.07 ± 10.21; education level 10.60 ± 3.64, AAT score: 1–3); 28 with moderate aphasia (18 males and 10 females; age 63.75 ± 8.36; education level 13.11 ± 3.98; AAT score: 4–5), and 15 with mild aphasia (10 males and 5 females; age 64.87 ± 12.08; education level 12.13 ± 3.40; AAT score: 6–7). Both the groups (PWA vs. control) were first contacted via telephone or e-mail, and those patients who gave their informed consent to the study were invited to take part in the survey. The online survey took around 20 min to be completed. The questionnaires were administered online to healthy people through forums and social network communities. In order to ensure that PWA could be assisted during the survey, the questionnaires were administered to the patients through Skype, a telecommunication platform specialized in providing video-chat and voice calls. The survey was anonymous and data confidentiality was assured. Depressive symptoms and anxiety were measured through the hospital anxiety and depression scale (HADS). Both the subscales consist of seven items scored on a 4-point Likert scale (0–3), yielding a maximum score of 21. A cut-off score = 8 was established to indicate pathological symptoms of depression or anxiety (Aben et al., 2002; Bjelland et al., 2002). The HADS scales have good internal consistency, good to excellent sensitivity and specificity, and good to very good concurrent validity (Aben et al., 2002; Bjelland et al., 2002). We used the stroke and aphasia quality of life scale (SAQOL-39), an interview-administered self-report scale to measure general dimensions of stroke-specific, health-related quality of life. The SAQOL-39 was validated for use in people with and without aphasia (Hilari et al., 2003, 2009). The questionnaire was comprised of 39 items grouped into 4 subdomains: physical, psychosocial, communication, and energy, which are the most affected areas in post-stroke patients (Parr et al., 1997; Duncan et al., 1999; Williams et al., 1999). The SAQOL has two response formats, both based on a 5-point scale: 1-could not do it at all to 5-no trouble at all and 1-definitely yes to 5-definitely no. Overall, subdomain scores can range from 1 to 5. The overall SAQOL score is calculated by adding across the items and dividing by the number of items; subdomain scores are calculated in the same way. Higher scores indicate a higher quality of life. The questionnaire has high validity and reliability in aphasic individuals and it is sensitive to changes (Hilari et al., 2003, 2009). Each questionnaire included two protocols, which were administered in a counterbalanced order across the groups. For each protocol, the questions were the same but they compared the psychological state of each participant between two distinct time periods: one month before (T0) and one month after the lockdown (T1).

### Data Analyses

All statistical analyses were conducted with IBM SPSS Statistics 22 software. For each group (PWA vs. control), paired *t*-tests were calculated on the mean score differences between the two time points: pre (T0)- and during (T1) COVID-19 in the different questionnaires. Two separate mixed ANOVAs were also performed for each subscale (anxiety vs. depression) of the HADS questionnaire with group as the between-subjects variable (PWA vs. control) and time as the within-subjects variable (T1 vs. T0). The primary analysis was the group-by-time interaction. The values of *p* < 0.05 were considered statistically significant. In both groups, correlational analyses (Pearson’s *r*-coefficient) were also conducted on the mean score differences among the different subscales between the two time points (T1 vs. T0).

To evaluate the impact of aphasia severity on the PWA’s responses in the different questionnaires, separate mixed model ANOVAs were also performed. We also analyzed the influence of gender, age, and educational level on the results collected in the two groups through two samples *t-*test comparisons (gender), the Kruskal–Wallis test (age), and a mixed-model ANOVA (educational level).

For each variable, in order to evaluate its statistical power, the values of the effect size were entered using the partial η2 index and the observed power index which SPSS software automatically associates with the ANOVA.

Our sample size was determined with the G^∗^Power 3.1.9.2 software (Faul et al., 2009). It was calculated that a sample size of at least 54 subjects per group was required to obtain an expected effect size of *d* = 0.51 (at 80% power; α = 0.05). Therefore, our samples were considered large enough to generalize the results to the reference population.

## Results

### Anxiety and Depression Scales

**FIGURE 1:**
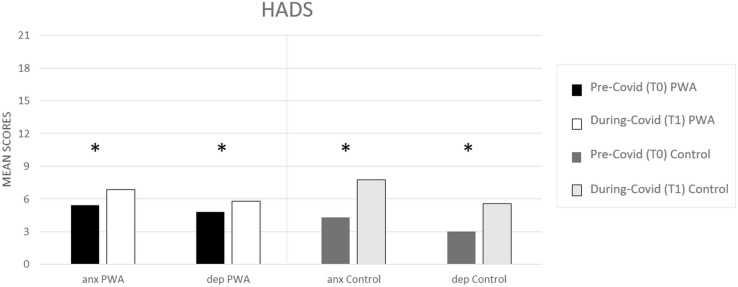
HADS mean scores (pre- and during COVID-19) for anxiety (anx) and depression (dep) scales, respectively, in the PWA and control groups. Sig. two tailed paired *t*-tests: ^∗^*p* ≤ 0.05.

In both groups, the prevalence of depressive symptoms and anxiety increased significantly between T0 (pre) and T1 (during COVID-19) (PWA: T0 5 vs. T1 = 6, *p* = 0.000 and T0 5 vs. T1 = 7, *p* = 0.000, respectively, for depression and anxiety; controls: T0 3 vs. T1 = 6 *p* = 0.000 and T0 4 vs. T1 = 8, *p* = 0.000, respectively, for depression and anxiety) (see Figure 1) as revealed by the paired *t*-tests.

**FIGURE 2:**
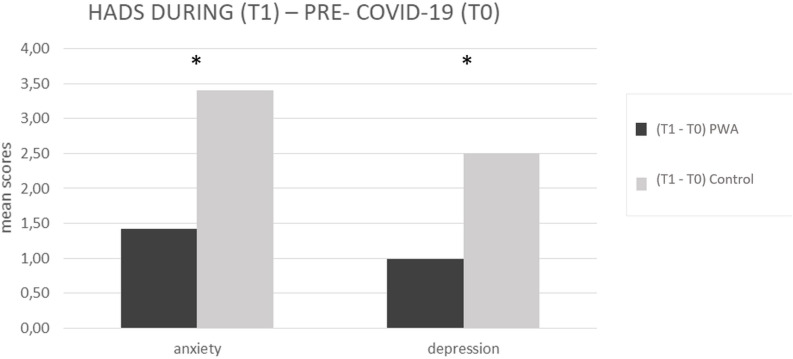
HADS mean scores difference between during (T1) and pre-COVID-19 (T0) for anxiety and depression scales, respectively, in the PWA and control groups. Sig. mixed ANOVA design – interaction time × group: ^∗^*p* ≤ 0.05.

The mixed ANOVA design performed on the anxiety levels of the HADS questionnaire revealed a significant effect of time (T1 vs. T0) [*F*(1,152) = 107.5, *p* < 0.001, partial η2 = 0.414, and observed power = 1.000] and no significant effect of group (PWA vs. control) [*F*(1,152) = 0.046, *p* = 0.831, partial η2 = 0.000, and observed power = 0.055]. The interaction time × group was also significant [*F*(1,152) = 18.1, *p* < 0.001, partial η2 = 0.106, and observed power = 0.988] showing that the anxiety levels were greater in the control group than in the PWA (T1–T0 controls = 4 vs. T1–T0 PWA = 2, *p* < 0.001) resulting above the cut-off score (Aben et al., 2002; Bjelland et al., 2002) (see Figure 2).

The mixed ANOVA design performed on the depression levels of the HADS questionnaire revealed a significant effect of time (T1 vs. T0) [*F*(1,152) = 73.8, *p* < 0.001, partial η2 = 0.327, and observed power = 1.000] and a significant effect of group [*F*(1,152) = 4.5, *p* = 0.035, partial η2 = 0.029, and observed power = 0.562]. The interaction time^∗^group was also significant [*F*(1,152) = 13.9, *p* < 0.001, partial η2 = 0.84, and observed power = 0.960] showing that the depression levels were greater in the control group than in the PWA (T1–T0 controls = 3 vs. T1–T0 PWA = 1, *p* < 0.001) (see Figure 2).

### SAQOL-39 Scale

**FIGURE 3:**
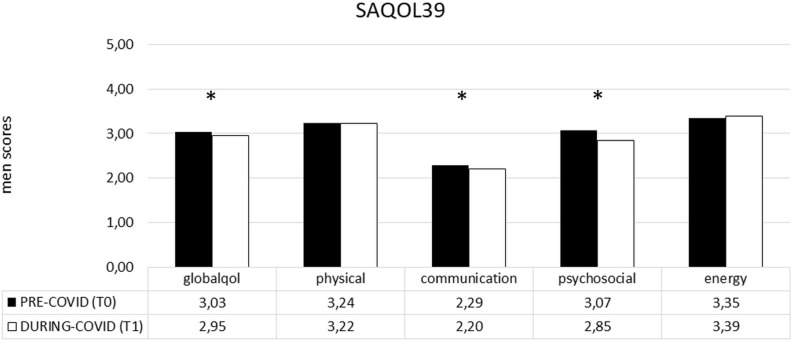
SAQOL-39 mean scores (pre- and during COVID-19) in the PWA group. Sig. two tailed paired *t*-tests: ^∗^*p* ≤ 0.05. Legend: globalqol, mean score in SAQOL-39 total score; physical, mean score in physical scale; communication, mean score in communication scale; psychosocial, mean score in psychosocial scale; energy, mean score in energy scale.

In the PWA group, the difference between the two time points (T1–T0) revealed that COVID-19 significantly impacted on the SAQOL-39 total score and, specifically on the communication and psychosocial domain. Indeed, the mean score was significantly lower at T1 than at T0, showing a significant deterioration of the performance in both areas (communication: T0 2,29 vs. T1 2,20 and *P* = 0.019; psychosocial: T0 3,07 vs. T1 2,85 and *P* = 0.001) (see Figure 3).

### Correlational Analyses

In both groups (PWA vs. control), the analyses (Pearson’s *r*-coefficient) performed on the mean percentage difference between the two time points (T1 vs. T0) revealed positive correlations (*p* ≥ 0.05) among the anxiety and depression scales (HADS) and the different subdomains of the SAQOL-39 scale. Only the psychosocial and the communication subdomains of the SAQOL-39 test were not correlated to each other.

### Influence of Aphasia Severity, Gender, Age, and Educational Level

Three separate mixed model ANOVAs performed with aphasia severity (severe – moderate – mild), as a between-subject factor, and time (T0–T1), as a within-subject factor, revealed that the detected changes between the two time points in the level of depression and anxiety of PWA and in the two subdomains of the SAQOL-39 questionnaire (communication and psychosocial area) were not significantly affected by the degree of aphasia severity. Similarly, when the variables, gender, age, and educational level were investigated, none of these factors significantly affected the scores in the different subscales.

However, the *post hoc* comparison between the three groups (severe vs. moderate vs. mild), showed that the severe group had a higher degree of anxiety and depression and worse performance in the different subscales of the SAQOL-39 questionnaire than the other two groups (moderate and mild), which differed from each only in the communication scale (see Table 1).

**TABLE 1:** Bonferroni *post hoc* comparisons between the three aphasic groups (severe vs. moderate vs. mild).

**Variable**	**Groups comparison**	***p*-value**
Hads-anxiety scale	Severe vs. moderate	0.001
	Severe vs. mild	0.015
Hads-depression scale	Severe vs. moderate	0.008
	Severe vs. mild	0.041
SAQOL-39 – total score	Severe vs. moderate	0.000
	Severe vs. mild	0.000
SAQOL-39 – physical	Severe vs. moderate	0.000
	Severe vs. mild	0.000
SAQOL-39 – communication	Severe vs. moderate	0.014
	Severe vs. mild	0.000
	Moderate vs. mild	0.000
SAQOL-39 – psychosocial	Severe vs. moderate	0.010
	Severe vs. mild	0.000
SAQOL-39 – energy	Severe vs. moderate	0.009
	Severe vs. mild	0.006

## Discussion

The main aim of the present study was to investigate the impact of the COVID-19 outbreak on different psychosocial dimensions, such as depression, anxiety, communication, and social isolation in persons with chronic post-stroke aphasia (PWA). A group of elderly matched controls also took part in the study. For the purpose of our work, it is necessary to highlight that all patients were already discharged from rehabilitation services before COVID-19 and they all lived at home with a caregiver.

Overall, results indicated that during the COVID-19 pandemic, both groups (PWA vs. control) scored significantly higher on the anxiety and depression subscales of the HADS questionnaire compared to the pre-COVID-19 condition. Moreover, although before the pandemic, the control group performed in the normal range with respect to the aphasic population, during the coronavirus, they reached significantly higher levels in both subscales, which even exceeded the cut-off score of the anxiety scale.

Thus, in line with several recently published studies, our work confirms that the COVID-19 emergency has dramatically affected the emotional state of the healthy population leading to an increase of depressive and anxiety symptoms (Brooks et al., 2020; Kang et al., 2020; Li et al., 2020; Moccia et al., 2020; Santini et al., 2020). It has already been suggested that the distorted perception of risk with fear and uncertainty experienced during the COVID-19 outbreak, along with the separation from loved ones and the limitations on freedom, have made a strong impact on mental health with an increase of negative emotions (Brooks et al., 2020). Accordingly, our healthy group reported emotional distress, low mood with depression, and anxiety. However, surprisingly, lower rates of depression and anxiety were found in PWA compared to the control group. These results, which, at first glance, may seem positive, actually mask a dramatic situation which affects aphasic people. Indeed, as stated in the Introduction, the anxiety and the depressive symptoms associated with poor social functioning, low quality of life, and poor functional abilities are frequently reported conditions in aphasic stroke survivors in the long term (Aström, 1996; Robinson, 1997; D’Alisa et al., 2005; Hilari et al., 2012; Jeong et al., 2012; Campbell Burton et al., 2013; Tang et al., 2013; Eccles et al., 2017). Normally, negative emotions are more pervasive in aphasic people than in healthy subjects, since their exclusion from language-dependent activities dramatically impacts their emotional wellbeing and social life (Engelter et al., 2006; Spaccavento et al., 2014; Baker et al., 2018; Mattioli, 2019; Pompon et al., 2019). In particular, as already stated, the patient tends to isolate his/herself and to have less intrinsic motivation in taking part in social relationships since he/she feels oppressed by negative symptoms (Cott et al., 2007; Woodman et al., 2014; Foley et al., 2019; Hjelle et al., 2019).

Thus, given that aphasic people already live in a state of social isolation, the virus had a paradoxically greater psychological impact on the control group (Spaccavento et al., 2014; Kirkevold et al., 2018). However, it is worth noting that the levels of anxiety and depression during COVID-19 have worsened in the aphasic population too. More importantly, since we also found a deterioration of their performance on the communication and psychosocial scales of the SAQOL-39 questionnaire which was positively correlated with their emotional status, we cannot exclude the possibility that these patients, already discharged from rehabilitation services, manifested a relapse on their communication skills due to the worsening of their psychological symptoms. Given these results, we believe that we must take into serious consideration not only the risks of COVID-19 on healthy people but also its impact on a population that is no longer benefiting from rehabilitation services. In fact, unlike what is often suggested by the literature (Thomas and Lincoln, 2008; Hilari and Byng, 2009; Hilari et al., 2012), we found no specific influence of aphasia severity with respect to the changes observed in anxiety, depression, communication, and psychosocial wellbeing. In other words, although there is a general agreement that aphasia severity correlates with patient performance (Thomas and Lincoln, 2008; Hilari and Byng, 2009; Hilari et al., 2012), in our study, the highest levels of anxiety and depression and the lowest rates in the communicative and psychosocial scales were independent of aphasia severity. Thus, COVID-19 had equally affected our three selected groups of aphasics. Similarly, no influences were found among the different demographic variables taken into consideration (educational level, age, and gender).

Given the current dramatic impact of COVID-19 on clinical services, although our aphasic population was already discharged from rehabilitation, our results point to the urgency of implementing new strategies and possible interventions for them. A very recent study (Bersano and Pantoni, 2020) also confirmed this need for acute stroke patients who, due to COVID-19, suffered from a shortage of services and delays in time-dependent treatments and diagnostic work-up. In order to circumscribe the consequences of the COVID-19 pandemic, one possible strategy might be to set up tele-rehabilitation and home-based services, which have been already successfully implemented for aphasia rehabilitation (Zhou et al., 2018; Gerber et al., 2019; Chang and Boudier-Revéret, 2020) and to focus on specific rehabilitation programs which allow the patients, who are no longer followed-up in therapy, to keep training their communication skills, and avoiding further deterioration. This would not add excessive costs to the healthcare system since it could be easily supported by the patients’ families.

Before concluding, it is worth noting that one possible limitation of our study is that our aphasic population was not entirely representative of the stroke population. Thus, our results do not allow for an advance in any final conclusion regarding the impact of COVID-19 on post-stroke people in general. However, since we strongly believe that one of the most serious socially disabling consequences after stroke is aphasia which does not offer the opportunity, as in other post-stroke syndromes, to communicate remotely, our results point to the urgency of taking into serious consideration the dramatic impact that COVID-19 has had on these patients.

In conclusion, since global attention is currently focused on clinical populations who, prior to COVID-19 were followed-up in rehabilitation services, our results point to the need of also considering the already discharged patients. For this population, we recommend they access home-based remote services in order to promote adaptive behaviors, reduce negative feelings, and prevent further deterioration of their cognitive performances.

## Data Availability Statement

The data will be available upon request.

## Ethics Statement

Ethical review and approval was not required for the study on human participants in accordance with the local legislation, institutional requirements and the legislation governing the psychologist profession (L.56/89). Written informed consent to participate in the study was provided by the patients.

## Author Contributions

FP and PM designed the research and wrote the manuscript. FP, CR, and MC performed the research. MC, CR, and AG analyzed the data. PM and AG edited the manuscript. All authors contributed to the article and approved the submitted version.

## Conflict of Interest

The authors declare that the research was conducted in the absence of any commercial or financial relationships that could be construed as a potential conflict of interest.
